# Golgi-Associated Protein JAKMIP2 Is Linked to the Centrosome and Performs Microtubule-Related Functions

**DOI:** 10.3390/cells14242019

**Published:** 2025-12-18

**Authors:** Evgeniia Ulas, Ilya Brodsky, Petros Padaryan, Anton Burakov

**Affiliations:** 1 A.N. Belozersky Institute of Physico-Chemical Biology, Lomonosov Moscow State University, 119992 Moscow, Russia; evgeniya.ulas@gmail.com (E.U.); brodskiy-i@yandex.ru (I.B.); p.padaryan@gmail.com (P.P.); 2 Institute of Protein Research of the Russian Academy of Sciences, 142290 Pushchino, Russia; 3 Faculty of Bioengineering and Bioinformatics, Lomonosov Moscow State University, 119991 Moscow, Russia

**Keywords:** centrosome, JAKMIP2, MTOC, cell differentiation, cell cycle, molecular rearrangements

## Abstract

Microtubules are present in all eukaryotes, and their spatial organization in the cells depends on the function of microtubule-organizing centers (MTOCs). Various organelles may act in this capacity, including the centrosome, Golgi, nuclear envelope, endosomes, and others. The molecular mechanisms that facilitate microtubule nucleation and/or anchoring at MTOCs are diverse. Many proteins can participate in these processes while localized to different MTOCs—either simultaneously on several or alternately on each of them. Here we studied the Golgi-associated protein JAKMIP2 in various cells using the methods of fluorescent multichannel confocal microscopy with subsequent image analysis via our own algorithm, transfection with a genetic construct encoding a fused protein of interest, and microtubule recovery monitoring during nocodazole washout. We demonstrated for the first time that JAKMIP2 is present on centrosomes in various cells. We also found that its abundance at this location is dependent on the cell cycle stage. Furthermore, we showed that an excess of JAKMIP2 specifically impairs centrosome function as a MTOC. Finally, our data indicates that exogenous JAKMIP2 slows down centrosomal microtubule nucleation and may also affect their anchoring. Our findings make a new contribution to the existing knowledge of the molecular mechanisms of the centrosome’s function as a MTOC.

## 1. Introduction

Microtubules are an essential component of all eukaryotic cells, as they are necessary for the formation of the mitotic spindle during cell division, including so-called closed mitosis. In interphase cells, the architecture of the tubulin cytoskeleton is highly diverse and corresponds to the functional needs of specific cells, depending on the characteristics of their life activities. Many cell types are characterized by their own unique microtubule system morphology. The structure of this system, in turn, directly depends on the function of the microtubule-organizing centers (MTOCs), where the processes of nucleation and/or anchoring are localized.

Traditionally, the centrosome is considered the main MTOC of the cell. This organelle was described in the 19th century, long before microtubules themselves were discovered. However, in recent years, other MTOCs distinct from the centrosome have been intensively studied. In humans and other animals, at the earliest stages of development, during cleavage, when cell divisions rapidly follow one another, the primary task of the tubulin cytoskeleton is the formation of mitotic spindles, and the role of the primary microtubule organizer belongs exclusively to the centrosome. However, later during growth and development, in the course of differentiation, the centrosome can partially or fully delegate its microtubule-organizing functions to other organelles. Thus, in fibroblasts, the centrosome serves as the first MTOC and the Golgi as a second (reviewed in [1,2]). In motile neurons at early stages of their development, the microtubule organization is centrosomal [3], but in mature neurons, the microtubules can be nucleated and anchored at the Golgi [4,5], endosomes [6,7], or other microtubules [8,9]. In differentiated muscle cells, the majority of the microtubule minus-ends associate with the nuclear envelope and the Golgi membranes [10,11]. In intestinal epithelium cells, the microtubule minus-ends are anchored directly to the cell cortex at the apical surface of the cells [12], to adherens junctions [13], or to tight junctions [14,15]. Regarding epidermis, as keratinocytes mature, the centrosome retains its ability to nucleate microtubules but no longer anchors them, and microtubules concentrate at desmosomes [16,17]. In pancreatic β cells, microtubules are nucleated at the Golgi but display no tight connection to any endomembranes [18,19]. Thus, it is now a well-established fact that microtubule-organizing centers other than the centrosome are present in the differentiated cells of various tissues.

The molecular mechanisms that capture the protein complexes responsible for microtubule nucleation and anchoring to various MTOCs are highly diverse. Importantly, the proteins involved in these processes exhibit varying degrees of “loyalty” to a specific MTOC—some may be unique to particular MTOCs, while others may initially be present on one MTOC and then disappear from it and are tethered to another MTOC, or these proteins may even be present on both MTOCs simultaneously. Thus, in columnar epithelia cells ninein is released from the centrosome, translocated with the microtubules in a microtubule-dependent manner, and is responsible for the anchoring of microtubule minus-ends to the apical non-centrosomal sites [20,21]. While keratinocytes are differentiated, ninein is also delocalized from the centrosome and recruited to the cell cortex to form a non-centrosomal MTOC [16,17]. In this case, ninein can connect microtubules to desmosomes by binding to desmoplakin [16]. During myoblast differentiation, γ-tubulin and centrosomal γTuRCs-recruiting proteins such as pericentrin, ninein, and CDK5RAP2, are delocalized from centrosomes and are subsequently recruited to the nuclear envelope [11,22,23]. In differentiated muscle cells, pericentrin and AKAP450 are recruited to the nuclear envelope by the outer nuclear membrane protein nesprin-1 [24,25]. In this case, the scaffold protein AKAP6 anchors centrosomal proteins to the nuclear envelope, acting as an adaptor between nesprin-1α and pericentrin or AKAP450 [26], and also participates in the recruitment of ninein and CDK5RAP2 to the nuclear envelope [27]. Though much is already known about the mechanisms of different cell compartments’ functions as MTOCs, not all of the proteins that might contribute to the processes underlying microtubule organization at various MTOCs have been identified yet. We became interested in the Golgi-localized protein JAKMIP2 as an alleged participant in microtubule organization.

JAKMIP2, also known as NECC1 (neuroendocrine long coiled-coil protein 1), is a member of three related proteins called JAKMIPs (Janus Kinase and Microtubule Interacting Proteins), which are highly conserved in vertebrates [28]. Endogenous JAKMIP2 is a peripheral membrane protein mainly localized on both the cis- and trans-Golgi in different neuroendocrine cell lines. Overexpression of exogenous JAKMIP2 in neuroendocrine cells leads to loss of Golgi compactness and to JAKMIP2 accumulation in juxtanuclear aggregates, localized around the centrosome, and presumably in secretory granules. Changes in JAKMIP2 level affect K-stimulated hormone secretion in neuroendocrine cells, supporting the idea that JAKMIP2 acts as a negative modulator of the regulated transport of secretory vesicles [29].

When studying proteins that might contribute to providing MTOCs’ functions, a good starting point for researching a specific protein is bioinformatic and biochemical data on interactions between that protein and its partners. In this regard, of particular interest is the fact that according to the published proteome-scale map, JAKMIP2 is predicted to interact with several proteins, among which is CLASP1 [30]. CLASP1 is a member of microtubule-associated proteins that decorates microtubule plus-ends both in the interphase [31,32] and mitosis [33], and regulates dynamics of microtubules [32,33], in particular stabilizing them by promoting pauses and restricting microtubule growth and shortening episodes [32]. However, protein distribution within the cell is not limited by its presence at the microtubule plus-ends only, and its role concerning microtubules extends beyond just their dynamic regulation. CLASP1 is also detected at the Golgi cisternae in retinal pigment epithelial cells and participates in microtubule nucleation processes at the Golgi membranes [34,35], thus being involved in providing Golgi apparatus functioning as MTOC. In addition, in HeLa cells, CLASP1 demonstrates localization on the centrosome (which is considered the main MTOC in many cell types) throughout interphase and mitosis [33,36,37]. As JAKMIP2, which probably interacts with CLASP1, was previously revealed at the Golgi network, but only in neuroendocrine cell lines [29], we decided to carefully examine the intracellular distribution of JAKMIP2 protein in cells of various human and animal lines and to examine its probable participation in microtubule organization processes.

## 2. Materials and Methods

### 2.1. Cell Cultures

U2OS (human osteosarcoma) cells were kindly provided by Dr. I. Terenin, HuH7 (human hepatocellular carcinoma) cells were kindly provided by Dr. V. Dugina, and fibroblast-like cultured green monkey kidney COS1 cells were taken from the lab stocks. All cells belong to the cell culture collections located in the laboratories of the specified researchers; the exact passage number of the cells is not determined. The cells were cultured in DMEM/F12 (1:1) culture medium (PanEco, Moscow, Russia) supplemented with 2 mM L-glutamine (PanEco, Moscow, Russia), 10% FBS (PanEco, Moscow, Russia), and 50 μg/mL gentamicin at +37 °C with 5% CO_2_.

### 2.2. Cell Fixation and Immunofluorescent Staining

The cells were fixed with 100% methanol for 7 min at −20 °C. Alternatively, the cells were fixed with 2% paraformaldehyde (PFA) in serum-free DMEM (with 20 mM HEPES and 5 mM MgCl_2_) for 10 min and then extracted with cold methanol (MeOH) for 5 min at −20 °C. The third method of cell fixation is when the cells were fixed with 100% methanol for 5 min at −20 °C and then with 3% paraformaldehyde (PFA) in PBS (20 min at +4 °C). For immunostaining, primary antibodies were used at 1–5 μg/mL and secondary antibodies were used at 5 μg/mL. The incubation time with both primary and secondary antibodies was 1 h at room temperature in the dark in a wet chamber. Primary mouse monoclonal antibodies against α-tubulin, clone DM1A (Sigma-Aldrich, Saint Louis, MO, USA; cat.# T9026), mouse monoclonal antibodies against JAKMIP2 (Santa Cruz Biotechnology, Dallas, TX, USA; cat.# sc-393578), rabbit polyclonal antibodies against γ-tubulin (kindly provided by Prof. R. Uzbekov), and rabbit polyclonal antibodies against ERGIC-53 (Santa Cruz Biotechnology, Dallas, TX, USA; cat.# sc-66880) were used. Mouse monoclonal antibodies (kindly provided by Dr. A. Minin) were the same as in [38]. As secondary antibodies, Alexa Fluor™ 568-conjugated anti-rabbit-IgG (Jackson ImmunoResearch Laboratories, Bar Harbor, ME, USA) and Cy5-conjugated anti-mouse-IgG (Jackson ImmunoResearch Laboratories, Bar Harbor, ME, USA) were used. Hoechst 33342 was kindly provided by Dr. S. Golyshev. The coverslips were mounted in Aqua PolyMount (Thermo Fisher Scientific, Waltham, MA, USA).

### 2.3. Microtubule Regrowth Experiments and Imaging

For microtubule disassembly, the cells were treated with a 3 μg/mL of nocodazole in culture medium (2 h at 37 °C, further 1 h at 0 °C, and finally 30 min at 37 °C). Then, nocodazole was washed out with warm cell culture medium, and the cells were incubated at 37 °C for the appropriate time prior to fixation.

For imaging, a Zeiss LSM900 confocal microscope was used (provided by the Moscow State University Development Program). The obtained data were processed using ZEN 3.5 blue edition software (Version 3.5.093.00002) (Zeiss, Oberkochen, Germany).

### 2.4. DNA Constructs and Liposomal Transfection

To obtain JAKMIP2 cDNA, total RNA was isolated from Vero cells using the RNeasy kit (74104, Qiagen, Hilden, Germany). First-strand cDNA was synthesized with Revert Aid reverse transcriptase (EP0441, Thermo Fisher Scientific, Waltham, MA, USA) and random hexanucleotide primers. JAKMIP2 cDNA was amplified with specific primers containing the terminal sites for restriction endonucleases (forward—ATAGGGCCCATGTCCAAGAAAGGGCGAAATA, reverse—ATAGGATCCCTTATCCATGTTTTCGGTTACTCTTTTC) and was further cloned into the pEGFP-C2 vector (Clontech, Mountain View, CA, USA). cDNA was verified by automated DNA sequencing. The CAMSAP3-GFP plasmid was the same as in [39].

High Fidelity PCR Enzyme Mix (K0191), thermosensitive calf intestinal alkaline phosphatase, and T4-ligase were from Thermo Fisher Scientific (Waltham, MA, USA). Restriction endonucleases were from Thermo Fisher Scientific (Waltham, MA, USA) or SibEnzyme (Novosibirsk, Russia). Primers were from either Syntol (Moscow, Russia) or Evrogen (Moscow, Russia). The Plasmid Miniprep Kit, PCR purification, and gel purification kits were from Evrogen (BC021, BC041S, Moscow, Russia) or Qiagen (12125, Germantown, MD, USA).

The transit-LT1 reagent (Mirus Bio, Madison, WI, USA) was used for cell transfection according to the manufacturer’s instructions.

### 2.5. Image Analysis and Statistical Analysis

To analyze the fluorescence level of centrosomes depicted in the summed Z-stacks, we developed a pipeline consisting of six stages: cell area separation by CellPose; finding the fluorescence maximum inside the cell mask in the γ-tubulin fluorescent channel (centriole centers); expanding the centrosome area; measuring protein fluorescence inside the centrosome area mask; filtering out false positives and adding false negatives (following protein fluorescence measurement); and characterizing cells by cell cycle stage (mitosis or interphase).

The first step was based on using CellPose [40]. We manually selected values for parameters. *Flow_threshold* was turned off (set to 0) as it defines the maximum allowed flow error for each mask, and we manually checked each one. A value of 0 was also assigned to both the *cellprob_threshold* (the threshold for flows to be treated as part of the ROI) and the *tile_norm_blocksize* (which computes normalization in tiles across the image to brighten dark areas. Mean diameter of the region of interest (ROI) was defined as 120 pixels (diameter = 120). Minimum size in pixels of supposed ROIs was defined as 20,000 (min_size = 20,000). *Niter*—number of iterations used to simulate a dynamical system governing pixel movement—was set to 2000, as recommended by the developers for large areas. We also enabled the *augment* parameter (set to True). It tiles the image with overlapping tiles and flips overlapped regions to augment. After cell areas were defined, the centrosome detection step began. First, the γ-tubulin fluorescent image was converted into grayscale and blurred by the GaussianBlur function to reduce small debris and noise artifacts. Then the blurred image was separated into cell masks, and inside each zone, one to two fluorescent peaks were identified. These two peaks were treated as centrioles. The peak searching function was restricted to a minimum distance of 6 pixels between peaks. Then centriole areas were expanded by including those pixels whose brightness was equal to or greater than 0.70 × peak_brightness. This threshold value was selected manually. We further analyzed the location of each peak by estimating the distance in pixels between them, and if it was less than 10 pixels, these two values were considered to be part of one centrosome, and the respective regions were merged. These regions were used as masks for protein fluorescence measurements. The respective fluorescence image was converted to grayscale, and the measurement of mean fluorescence inside each mask was performed. Each area was also filtered by size, and if it was too small (less than 30 pixels), it was treated as noise and removed.

To filter out false positives and identify false negative cases (cells lacking detected centrosomes), each detected centrosome region was visualized on the corresponding image and labeled as *cell_number–centrosome_number*. This manual inspection confirmed that the pipeline was generally sensitive: false negative cases were rare.

Statistical analysis was implemented using IBM SPSS Statistics, version 29.0.0.0 (241). For the purpose of correct comparison of JAKMIP2 protein amounts present on centrosomes in mitotic and interphase cells, the filtering-out stage was supplemented by the additional step so that only cells with two distinct centrosomes detected were used in the following statistical analyses. For statistical significance assessment of differences in JAKMIP2 fluorescence levels at centrosomes between cells in different cell cycle stages, the Random Intercept Model was applied to the resulting data sets, as it accounted for nested variation. The “stage of the cell cycle” (mitosis/interphase) was established as the fixed effect, and “JAKMIP2 protein fluorescence intensity at the centrosome” was established as the dependent variable. Based on the fact that each of the chosen cells contains two centrosomes, the possible variability in fluorescence intensity of the JAKMIP2 protein on each centrosome present between different cells due to their random individual differences was taken into account by defining the “cell number” as the random effect.

During measurements of the acetylated tubulin level, as the cell density in the images was high, CellPose failed to segment the cells. Therefore, this step was performed manually using the FIJI plugin. For this purpose, the freehand selection tool was used to determine cell areas. Appropriate boundaries were drawn on images with visualized tubulin, chromatin, and the expressed protein construct. After each ROI (presumed cell) was added to the ROI Manager, mean brightness was measured by the built-in ROI Manager measurement tool on the images with visualized tubulin only. All data were compiled into a single dataset, which also contained the following columns: *image* (image number), *cell_id* (unique cell identifier within each image), and *transfected* (True if the transfection succeeded in the cell; otherwise, False).

To assess the statistical significance of the differences in acetylated tubulin level within microtubules between control and transfected cells, the Mann–Whitney U-test was performed.

No generative artificial intelligence (GenAI) has been used in this paper to generate text, data, or graphs, or to assist in study design, data collection, analysis, or interpretation.

## 3. Results

### 3.1. Endogenous JAKMIP2 Is Localized to Centrosomes in Various Cell Lines

It was previously shown that endogenous JAKMIP2 is a peripheral membrane protein localized to both the cis- and trans-Golgi in neuroendocrine cells (see Introduction above). Since JAKMIP2 is predicted to interact with CLASP1, a protein that demonstrates centrosomal localization throughout interphase and mitosis at least in some cell types, we decided to first examine the intracellular localization of JAKMIP2 in different cell lines of non-neuroendocrine origin.

**Figure 1:**
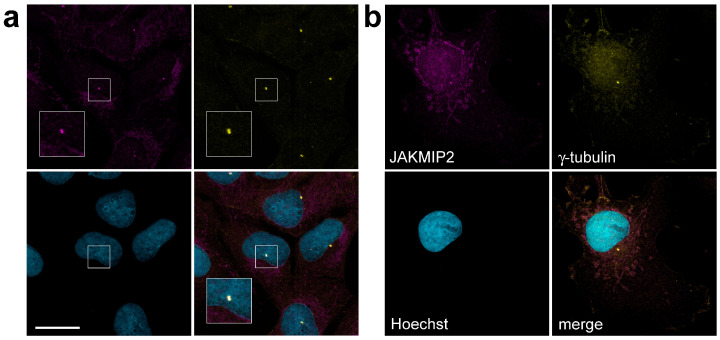
Immunofluorescent staining of U2OS and COS1 reveals a centrosomal pattern of JAKMIP2 localization. (**a**) Triple staining of U2OS cells with antibodies against JAKMIP2, γ-tubulin, and Hoechst 33342. The area in the square is shown enlarged below, demonstrating the co-localization of JAKMIP2 and γ-tubulin at the centrosome. The scale bar is 20 μm for both a and b panels. (**b**) The same staining of COS1 cells shows a similar picture.

We performed immunofluorescence staining of various cell lines with antibodies against JAKMIP2 and found that they indeed stained a pattern morphologically very similar to the Golgi cisternae, which is fully consistent with previously obtained data. However, we also found that these antibodies additionally stained characteristic small bright dots located in the cytoplasm (Figure 1). This pattern was observed in human osteosarcoma U2OS cells (Figure 1a), human hepatocellular carcinoma HuH7 cells (Supplemental Figure S1), and fibroblast-like COS1 cells derived from African green monkey kidney cells (Figure 1b). 

Double staining of these cells with antibodies against JAKMIP2 and a marker centrosomal protein γ-tubulin, associated with pericentriolar material [41] and also being identified as part of centrioles [42], demonstrated a marked co-localization of the signals. We used several different cell fixation methods (see the Section 2 above) and observed identical results regarding the centrosomal localization of the JAKMIP2 protein. We opted for methanol fixation, as this fixation method clearly distinguishes both localization patterns of JAKMIP2—on the intracellular structures very similar to the Golgi and on the centrosome. When using paraformaldehyde in addition to methanol, the fluorescent signal of JAKMIP2 at the centrosomeswas the same, but background staining was observed in the nuclei, and the localization of JAKMIP2 on the Golgi was not detected (Supplemental Figure S2). Therefore, in Figure 1 and throughout the article, the results of methanol fixation are presented.

Thus, we have demonstrated that endogenous JAKMIP2 (Janus Kinase and Microtubule Interacting Protein 2), also known as NECC1 (Neuroendocrine Long Coiled-Coil Protein 1), is localized not only on the Golgi apparatus but also on centrosomes in several cultured cell lines.

### 3.2. The Amount of JAKMIP2 on Centrosomes Can Vary

**Figure 2:**
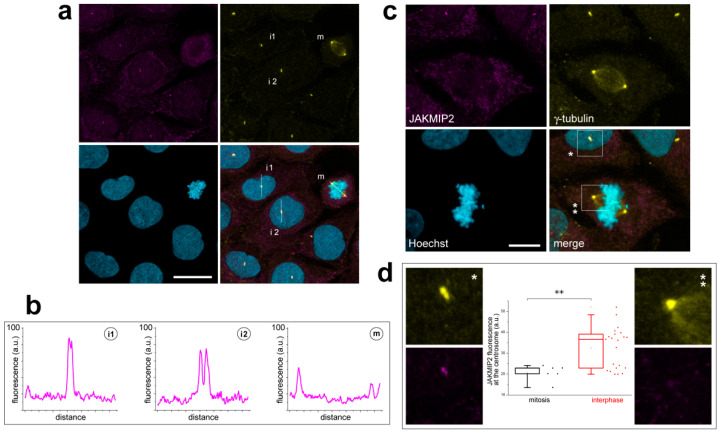
Triple staining of U2OS cells with anti-JAKMIP2, anti-γ-tubulin, and Hoechst 33342 demonstrates brighter JAKMIP2 staining of interphase centrosomes compared to mitotic ones. (**a**) Several interphase cells and a mitotic cell in a single field of view. Line scans were performed along the designated lines to measure fluorescence. (i)—interphase cell, m—mitotic cell. The two selected interphase cells differ in the distance between their centrioles. The scale bar is 20 μm. (**b**) JAKMIP2 fluorescence profiles along the lines indicated in panel (**a**). High fluorescence peaks in interphase cells and lower peaks in mitotic cells are clearly visible. (**c**) The amount of endogenous JAKMIP2 at the centrosomes is lower in mitotic cells (**) compared to interphase cells (*). The areas in the squares are shown enlarged in panel (**d**). The scale bar is 10 μm. (**d**) Enlarged interphase and mitotic centrosomes shown in (**c**), and fluorescence measurement statistics. For the initial selection of centriole images suitable for further analysis, 50 summed Z-stacks of individual cells were used (see Section 2 and text below), ** *p* < 0.008.

Image analysis of co-staining with antibodies against JAKMIP2 and γ-tubulin suggested that mitotic centrosomes, located at the spindle poles, are stained more weakly for JAKMIP2 than interphase ones. This is despite the fact that the functional activity of centrosomes as MTOCs is higher during mitosis, as confirmed by their brighter staining with γ-tubulin antibodies. Measurements of JAKMIP2 antibody fluorescence intensity along line scans drawn through the centrosomes confirmed this observation (Figure 2a,b).

In interphase cells, we observed a sharp, high fluorescence peak in the centrosomal region that exhibited a double-hump pattern, which is more or less pronounced depending on the distance between the centrioles. In contrast, a line scan drawn through both spindle poles in dividing cells revealed much lower fluorescence peaks in the centriolar regions, with one of the centrosomes always being noticeably dimmer than the other (Figure 2a,b).

We performed a detailed statistical analysis of fluorescence measurements on centrosomes, both manually and using a custom-developed software algorithm (see the Section 2 above), which fully confirmed these observations. Indeed, the fluorescence intensity at mitotic centrosomes is approximately halved compared to interphase ones (Figure 2c,d). Furthermore, the fluorescence at one of the mitotic spindle pole centrosomes often disappears almost completely.

The obtained z-stacks were filtered out to comply with the requirements described in Section 2 for performing statistical analyses. Initially, 64 JAKMIP2 fluorescence peak measurements were defined as 65 centrosomes belonging to 50 different cells at the first step of z-stack data processing. Filtering procedure application kept 28 fluorescent peaks as 28 centrosomes belonging to 14 cells for the following statistical analyses. In particular, from the original data set of 5 mitotic cells containing 8 fluorescent peaks, 2 cells containing only one fluorescent peak were removed, resulting in 3 cells containing 6 fluorescent peaks. Regarding interphase, from the initially detected 45 cells with a total amount of 56 fluorescent peaks, only 11 cells containing 22 fluorescent peaks were selected. The final data sets were suited for the usage of the Random Intercept Model as the precise method of statistical analysis. The difference in the amount of JAKMIP2 protein on centrosomes in interphase and mitotic cells is statistically significant, since the *p* value is 0.008, which is indicated on the graph by two asterisks (Figure 2d).

Thus, we have demonstrated that endogenous centrosomal JAKMIP2 tends to dissociate from the centrioles as cells enter mitosis. Given the primary function of the centrosome, we next started to determine whether JAKMIP2 is involved in this process. This was of particular interest given that its possible interacting partner, CLASP1, is involved in the same processes (see Introduction). To investigate the functions of our protein of interest, we decided to overexpress fluorescently tagged JAKMIP2 in cells.

### 3.3. Exogenous JAKMIP2 Tends to Accumulate in the Centrosomal Region

**Figure 3:**
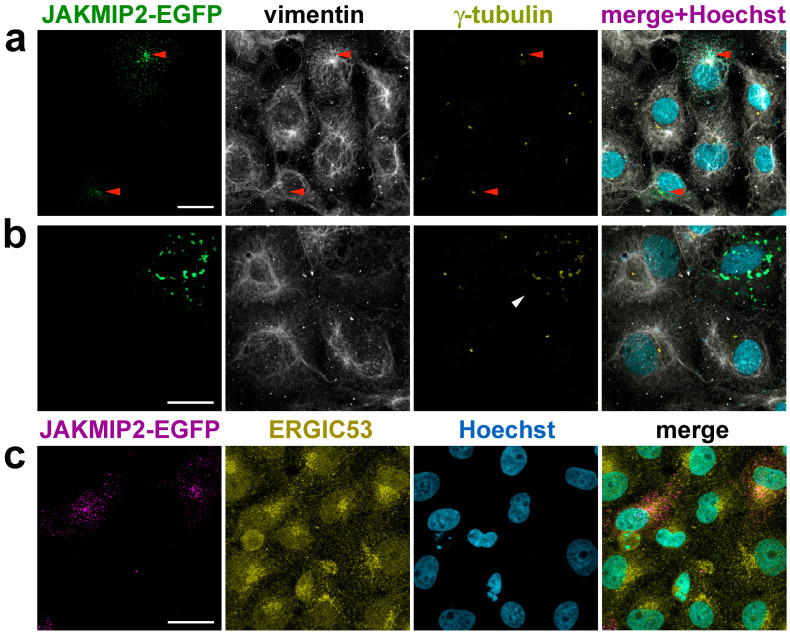
Accumulation of exogenous JAKMIP2 in the centrosome region does not affect the morphology of vimentin intermediate filaments. (**a**) Co-staining with antibodies to vimentin and γ-tubulin of COS1 cells expressing JAKMIP2-EGFP at low levels demonstrates small JAKMIP2-positive dots that tend to cluster in the pericentrosomal region (the centrosome is marked with red arrows). The organization of vimentin filaments in transfected cells is indistinguishable from that in control cells. The scale bar is 20 μm; the same applies to panels (**b**,**c**). (**b**) Overexpression of JAKMIP2-EGFP can lead to the formation of aggresomes in the perinuclear region of the cell, which disrupts the morphology of vimentin intermediate filaments. The large clumps of exogenous JAKMIP2 are positively stained with antibodies against γ-tubulin (marked with a white arrow). (**c**) The presence of exogenous JAKMIP2 does not affect Golgi compactness in transfected cells, as confirmed by staining with antibodies against ERGIC-53, a marker of the ER-Golgi intermediate compartment.

It was previously shown by other authors that overexpression of JAKMIP2 in neuroendocrine cells leads to exogenous protein accumulation in juxtanuclear aggregates, localized around the centrosome, and presumably in secretory granules [29]. For our work, we chose easily transfectable fibroblast-like COS1 cells, which possess a well-defined radial system of centrosomal microtubules. Expression of JAKMIP2-EGFP in these cells demonstrated that the exogenous protein is distributed throughout the cell as small dots, with a tendency to accumulate in the centrosomal region, the location of which we detected by γ-tubulin staining, consistent with previously published data (Figure 3a, indicated by red arrows). It is known that, unlike microtubules, the organization of intermediate filaments is not linked to the centrosome, so the location of centrioles does not, as expected, coincide with the areas of the densest vimentin filaments.

Before proceeding to study the effect of exogenous JAKMIP2 on cellular functions, we carefully verified that the observed puncta were not aggressomes. Aggresomes are defined as a pericentrosomal accumulation of aggregated, insoluble proteins that can be derived from misfolded or overexpressed proteins [43,44]. Their presence, in particular, disrupts the normal astral distribution of microtubules [44] and also suppresses microtubule nucleation at the centrosome [45]. It was previously shown by other authors that the presence of aggresomes also affects the spatial organization of vimentin intermediate filaments [43,44]. Therefore, we stained transfected cells with vimentin antibodies and found that at low levels of exogenous JAKMIP2 expression, the vimentin intermediate filaments in transfected cells were indistinguishable from those in control cells (Figure 3a). Therefore, the observed small dots were not aggresomes.

Conversely, during strong overexpression, exogenous JAKMIP2 formed large aggregates in the juxtanuclear area of the cell, and in this case, the vimentin intermediate filament system was drastically different from that in adjacent non-transfected cells (Figure 3b). This suggests that such aggregates indeed likely were aggresomes, and any observed changes in intracellular processes in these cells were artificial. Interestingly, we found that these large aggregates of exogenous JAKMIP2 were stained by the γ-tubulin antibodies we used (Figure 3b, white arrow), providing a method to exclude these unsuitable transfected cells from analysis even without vimentin staining. Subsequently, all morphometric analyses were performed using only cells with low levels of exogenous JAKMIP2 expression.

We additionally investigated the effect of exogenous JAKMIP2 on the compactness of the Golgi, which we detected using antibodies against ERGIC-53 that marked the ER-Golgi intermediate compartment. We observed no noticeable differences in Golgi compactness between transfected and control cells (Figure 3c). Overall, the distribution of the exogenous protein indicated an affinity for the centrosome rather than a close association with Golgi membranes. Furthermore, the compactness of the Golgi in transfected cells with low JAKMIP2-EGFP expression serves as additional evidence that the observed dots are not aggresomes, as it was previously shown that aggresomes interfere with correct Golgi localization [44].

Thus, we have shown that exogenous JAKMIP2-EGFP tends to accumulate in the centrosomal region. Additionally, we established criteria to exclude cells with excessively high JAKMIP2 expression, thus preventing incorrect conclusions. Following this, we proceeded to analyze what changes in the cytoskeleton are caused by the presence of additional exogenous JAKMIP2.

### 3.4. Elevated JAKMIP2 Levels Affect Tubulin Cytoskeleton and Reduce the Amount of Acetylated Microtubules

**Figure 4:**
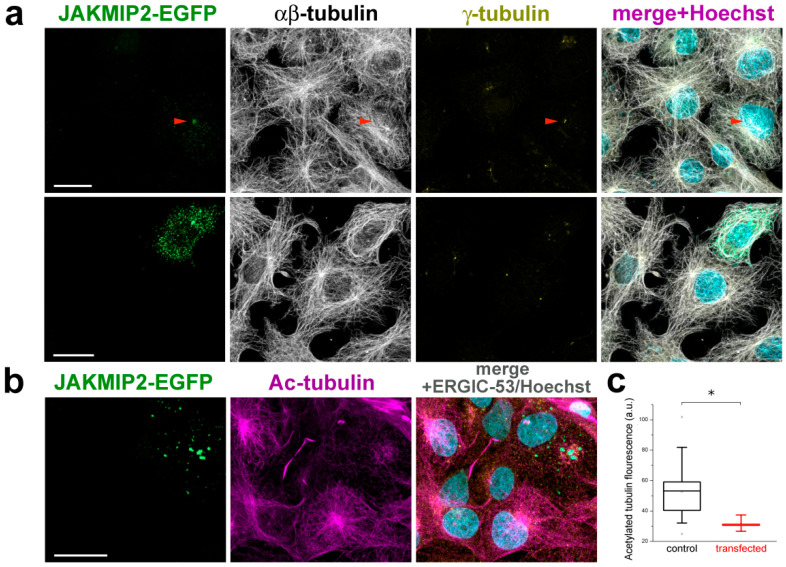
The presence of exogenous JAKMIP2 significantly affects the radiality of the microtubule network and reduces the amount of acetylated tubulin. (**a**) (Top panels) Co-staining of JAKMIP2-transfected COS1 cells with antibodies against α-tubulin and γ-tubulin demonstrated that accumulation of exogenous protein in the centrosome region reduces the radial arrangement of microtubules in the transfected cells. As a result, the centrosome ceases to perform the functions of the main MTOC of the cell (red arrow). (Bottom panels) As the amount of exogenous protein increases, the microtubules become completely chaotic. Note that the level of JAKMIP2 expression is still insufficient for aggresome formation, as confirmed by staining with antibodies against γ-tubulin. The scale bar is 20 μm for both (**a**,**b**). (**b**) Staining of transfected COS1 cells with antibodies against acetylated tubulin shows that JAKMIP2-EGFP-positive cells contain fewer stabilized microtubules. (**c**) Fluorescence measurement statistics for acetylated tubulin staining (see Section 2). The general data of two experiments are presented; statistical analysis was performed for 38 cells. * *p* < 0.05.

Since we found that exogenous JAKMIP2 often accumulates in the centrosomal region, we stained cells not only for γ-tubulin but also for αβ-tubulin to visualize the microtubule network responsible for intracellular transport towards and away from the centrosome. We discovered that even at the very lowest initial levels of exogenous protein expression, the radial structure of the microtubule system begins to deteriorate, becoming tangled as expression levels of JAKMIP2 increase (Figure 4a). It is evident that in all surrounding non-transfected cells, the centrosome is a clear focal point for converging radial microtubules, whereas even minimal presence of exogenous JAKMIP2-EGFP causes it to lose this role of the main MTOC (Figure 4a, red arrow). This phenomenon was observed in all transfected cells and was dependent on the level of JAKMIP2 expression. Line scan analysis across images of microtubule-stained cells initially showed a reduction and broadening of the tubulin fluorescence peak in the centrosome area, followed by its complete disappearance, as was observed when microtubule architecture was disturbed in the cell in another way [46,47]. Note the absence of γ-tubulin staining in transfected cells anywhere other than the centrosomes, indicating the specificity of the observed effect (compare with Figure 3b).

Another staining of transfected cells, with antibodies against acetylated tubulin, revealed a noticeable reduction (approximately 60% of the level in control cells) in stabilized acetylated microtubules, likely emanating from the centrosome (Figure 4b,c). The difference in the level of acetylated microtubules in control cells and cells expressing exogenous JAKMIP2 is statistically significant, since the *p* value is 0.05, which is indicated on the graph by the asterisk symbol (Figure 4c). This observation suggests that excess JAKMIP2 influences microtubule network morphology by affecting processes related to their dynamics. Therefore, we decided to investigate the nucleation of new microtubules at centrosomes.

### 3.5. Increased Levels of JAKMIP2 Slow Down the Rate of Microtubule Nucleation at Centrosomes

We decided to determine whether the observed loss of radial microtubule network structure was directly linked to the excess JAKMIP2 at centrosomes, which might interfere with its function as an MTOC. To test this, we performed microtubule regrowth assays during nocodazole washout and compared the dynamics of centrosomal microtubule asters formation in control and transfected cells.

**Figure 5:**
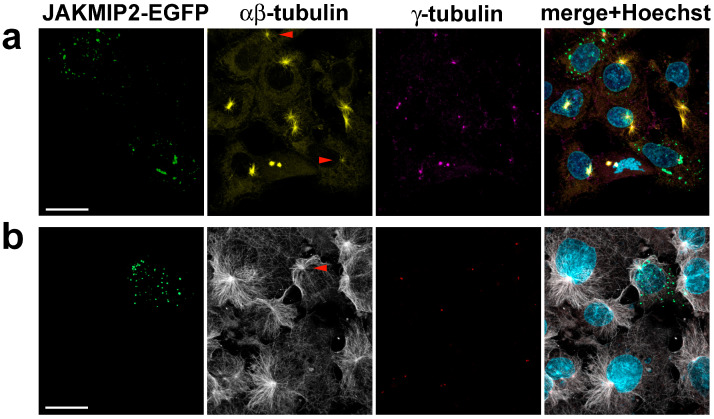
Microtubule nucleation at centrosomes is affected in cells containing exogenous JAKMIP2. (**a**) Early stages of nocodazole washout (2 min) show smaller centrosomal microtubule asters in transfected COS1 cells (red arrows) compared to control ones; this difference disappears at later stages (10 min of nocodazole washout) (**b**). Note the absence of non-specific staining of transfected COS1 cells with antibodies against γ-tubulin, indicating the absence of aggresomes in the cells with JAKMIP2-EGFP. The scale bar is 20 μm for both (**a**,**b**) panels.

We found that at the early stages of microtubule regrowth (2 min after nocodazole washout), centrosomes in transfected cells typically exhibited noticeably smaller asters compared to surrounding control cells (Figure 5a). In all cases, this was a consequence of excess exogenous JAKMIP2 and not due to aggresome formation, which we confirmed by immunofluorescence staining for γ-tubulin. However, the centrosomes in transfected cells retained their functionality as MTOCs, since at later stages of regrowth (10 min after nocodazole washout), the large centrosomal microtubule asters in transfected cells were indistinguishable from control ones (Figure 5b).

The observed pattern is markedly different from what we see during nocodazole washout in cells overexpressing CAMSAP3. It is known that this microtubule minus-end binding protein regulates Golgi assembly in epithelial cells by affecting microtubule dynamics [48]. At high levels of overexpression, CAMSAP3 causes all cellular microtubules to coalesce into thick, stable bundles that are nocodazole-resistant [39]. However, low expression levels result in only a few single CAMSAP-decorated stable microtubules in the cytoplasm, with the centrosomal microtubule aster being indistinguishable from that in control cells (Supplementary Figure S3). This indicates that the impact of exogenous JAKMIP2 on the centrosome is specific.

Thus, an excess of JAKMIP2 at centrosomes leads to an impairment of microtubule nucleation processes but does not completely abolish them. It is possible that anchoring processes are compromised, ultimately leading to the observed microtubule disorganization, but this hypothesis requires further investigation in future studies.

## 4. Discussion

In this study, we have demonstrated for the first time the centrosomal localization of the Golgi-associated protein JAKMIP2 in various cell types. We also showed that this localization is cell cycle-dependent: interphase centrosomes contain nearly twice as much of this protein as mitotic centrosomes. Furthermore, we demonstrated that an excess of JAKMIP2 alters microtubule-network organization and reduces the amount of stable acetylated microtubules in cells. Finally, we showed that excess JAKMIP2 negatively affects the process of microtubule nucleation at the centrosomes.

These new findings are consistent with each other. If JAKMIP2 plays a role in microtubule anchoring, since both nucleation and anchoring are spatially co-localized, excess protein due to its overexpression could sterically hinder the nucleation of new microtubules at this specific location. Given that cells need to sharply increase the nucleating activity of their centrosomes upon entering mitosis, it is logical that a protein whose excess can inhibit nucleation dissociates from centrosomes at mitotic entry.

Regarding the interpretation of data on differences in JAKMIP2 levels at centrosomes between interphase and mitotic cells, we can exclude the possibility that the JAKMIP2 present on the initial interphase centrosome is simply partitioned equally between the two daughter centrosomes during duplication in S-phase and the protein level on these centrosomes does not subsequently increase throughout mitosis. This is because we deliberately selected for statistical analysis only those interphase cells that already contained two centrosomes (as described in Materials and Methods). Therefore, our comparison of JAKMIP2 levels on centrosomes between interphase and mitotic cells is valid.

Previously, the functions of the JAKMIP2 protein were unknown, with the exception of the fact that it acts as a negative modulator of regulated secretory vesicle transport in neuroendocrine cells [29]. However, it was shown earlier that another member of the JAKMIP family, JAKMIP1 (also known as Marlin-1), interacts with microtubules [28] and kinesin I [49] by its N-terminal region containing multiple coiled-coil domains and is involved in the regulation of microtubule-network stability in fibroblasts [28]. The endogenous JAKMIP1 is also co-localized with the microtubule network in the lymphoid cell line Jurcat [28] and decorates microtubules in neurons, where it forms a complex with kinesin 1 and is involved in the intracellular transport of GABAB receptors [49]. It is also known that JAKMIP1 and JAKMIP2 are highly conserved in vertebrates [28] and share 58% identity in their amino acid sequences, with the highest similarity in N-terminal regions (but JAKMIP2 has a longer C-terminal region) [28,50]. Therefore, it is likely that JAKMIP2 may be able to interact with microtubules and affect their dynamics too [28]. These assumptions are supported by the prediction of its interaction with CLASP1 [30], the microtubule-associated protein that regulates microtubule dynamics [32,33] and is involved in the microtubule nucleation at the Golgi membranes [34,35]. In the present study, we have demonstrated that the functions of JAKMIP2 at the centrosome are associated with microtubule organization and, possibly, with their anchoring, as an excess of this protein at the centrosomes leads to a partial slowdown of nucleation, subsequently resulting in a reduced number of stable acetylated microtubules and disorganization of the entire microtubule network.

As for the proposed mechanism of JAKMIP2 recruitment to the centrosome, it might also be related to the predicted JAKMIP2 and CLASP1 interaction. It was previously reported that CLASP1 is localized both on the Golgi network [34,35], being bound by the Golgi membrane protein GCC185 [34], and on the centrosome [33,36,37], at least in some cell lines. The same intracellular distribution pattern was detected for JAKMIP2 in our study. There is a chance to speculate that JAKMIP2 might accumulate at specific compartments, such as the centrosome and Golgi membranes, by interacting with its possible partner, CLASP1.

It is quite possible that the functions of the centrosome-associated and Golgi-associated JAKMIP2 are not entirely identical. It is well known that the same protein can perform different functions when located in different cellular compartments. A prime example is cytoplasmic dynein, which acts as a motor protein transporting cargo along microtubules through the cytoplasm [51,52], while also being able to generate pulling forces on microtubules reaching the cell boundary when associated with the cell cortex [53]. Furthermore, when anchored at the centrosome, it participates in retaining the microtubule minus-ends [47]. That is why it is particularly interesting to investigate the functions of any protein when we discover it in a previously unknown cellular location. In this study, we have found JAKMIP2 in a new location—at the centrosome—and have shown that it somehow influencesthe centrosome in performing its microtubule-organizing functions. Regarding the endogenous JAKMIP2, at least two distinct isoforms of the protein are present in different cells, as JAKMIP2 mRNA is alternatively spliced in a tissue-specific manner [28,50]. As the antibodies against JAKMIP2 that we used to perform experiments for this article were unable to distinguish one isoform from another, it is even possible that these two isoforms of JAKMIP2 are distributed in different compartments of the cell and perform different functions.

## Data Availability

The original contributions presented in this study are included in the article. Further inquiries can be directed to the corresponding author.

## Supplementary Materials

The following supporting information can be downloaded at: https://www.mdpi.com/article/10.3390/cells14242019/s1, Supplementary Figure S1. Triple immunofluorescent staining of HuH7 cells with antibodies against JAKMIP2 and γ-tubulin, and with Hoechst 33342. Scale bar is 10 μm. Supplementary Figure S2. Triple immunofluorescent staining of U2OS cells fixed by PFA/methanol (see Section 2) with antibodies against JAKMIP2 and γ-tubulin, and with Hoechst 33342. Scale bar is 20 μm. Supplementary Figure S3. The nocodazole washout microtubule regrowth experiment in COS1 cells expressing low levels of CAMSAP3-GFP. Scale bar is 20 μm.

## Abbreviation

The following abbreviation is used in this manuscript:

MTOC	Microtubule-organizing center
