# Supplementary figures and images for: Golgi-Associated Protein JAKMIP2 Is Linked to the Centrosome and Performs Microtubule-Related Functions
Source: Cells. 2025 Dec 18;14(24):2019. doi: 10.3390/cells14242019 (PMC12732012; doi:10.3390/cells14242019)

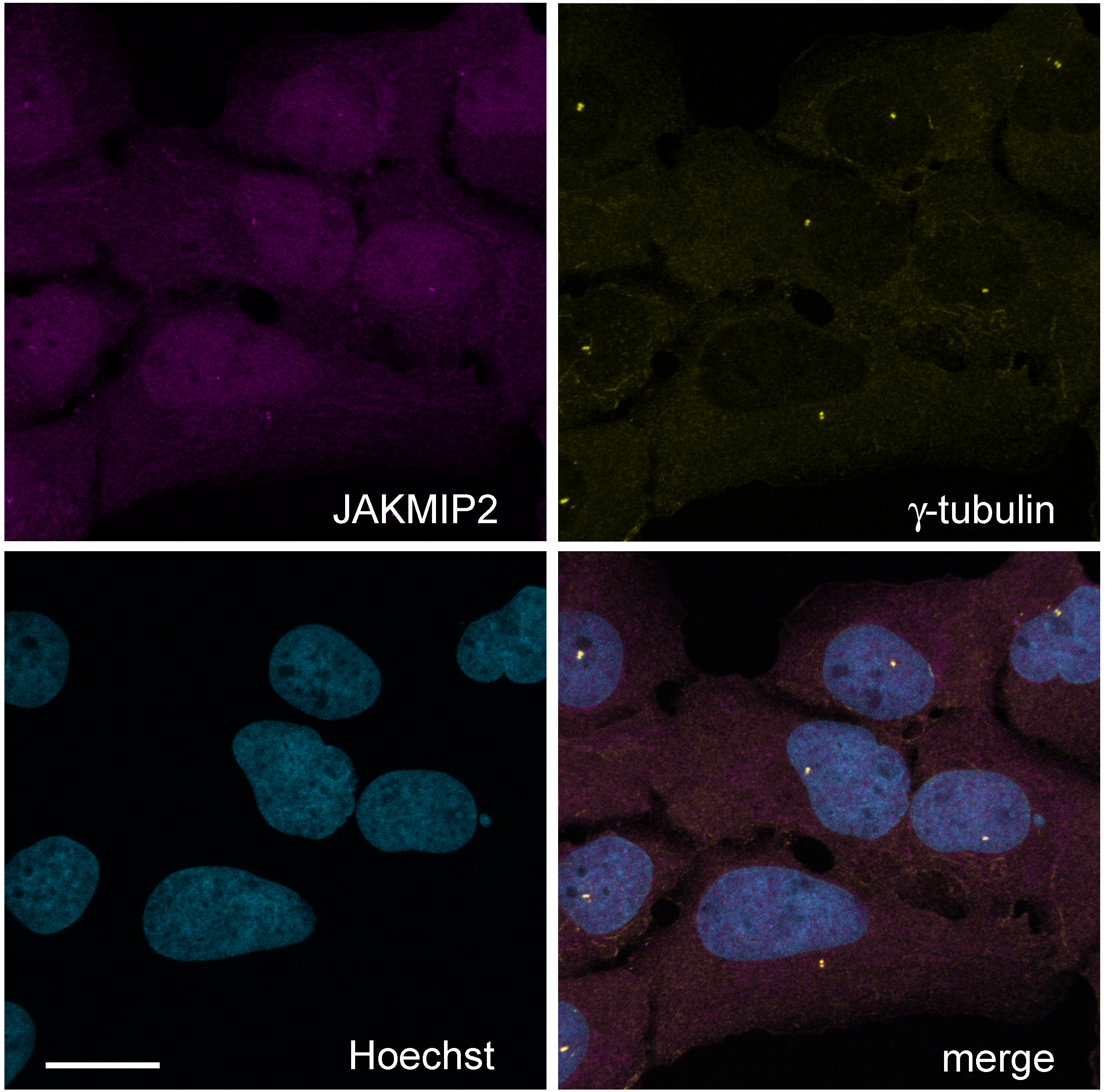

Supplement: Supplementary file 1 [file cells-14-02019-s001.zip › Supplemental Figure S2.tif]

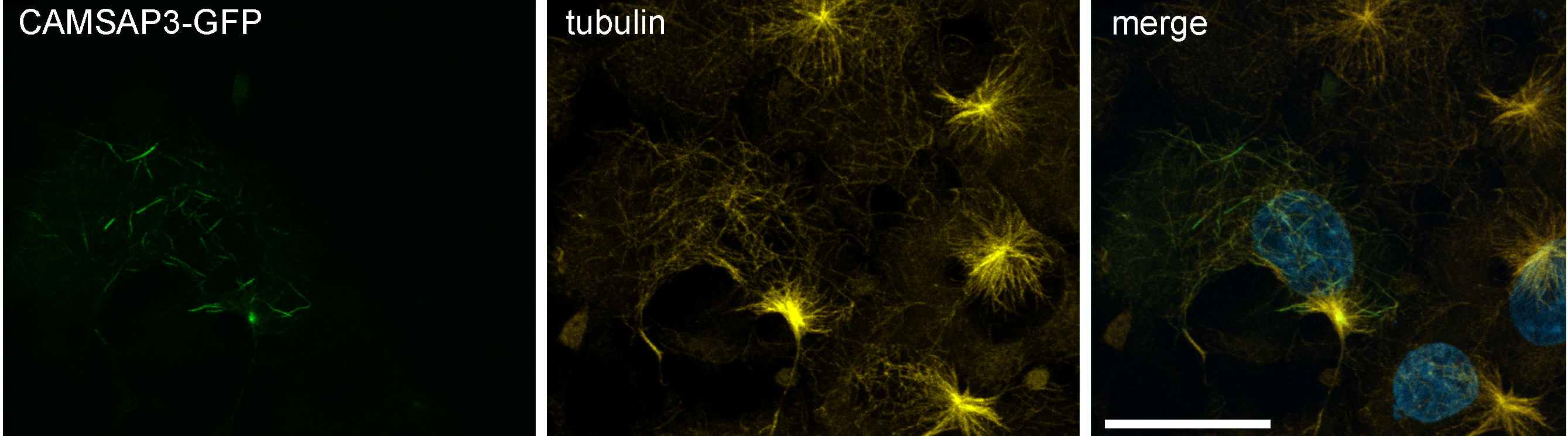

Supplement: Supplementary file 1 [file cells-14-02019-s001.zip › Supplemental Figure S3.tif]

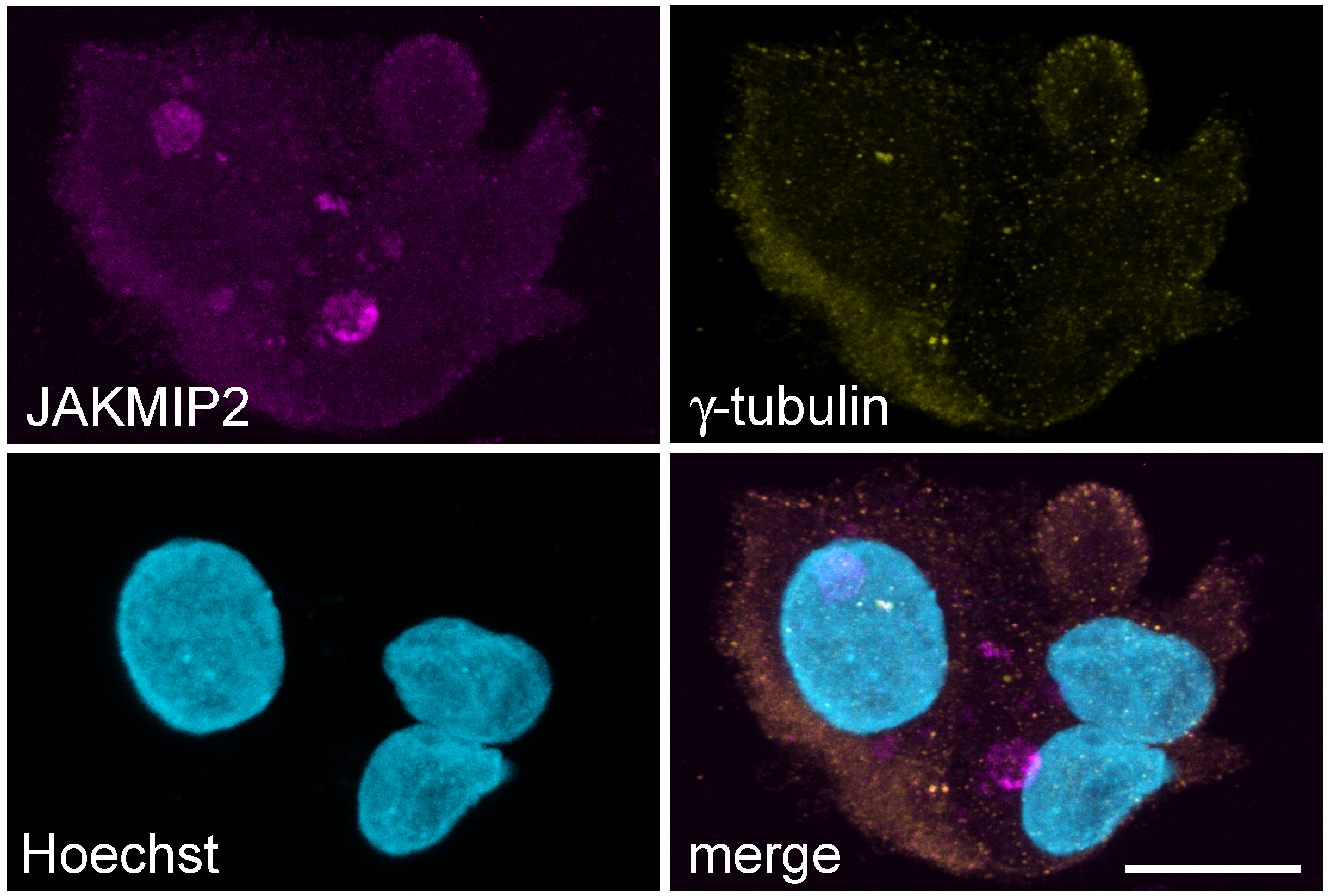

Supplement: Supplementary file 1 [file cells-14-02019-s001.zip › Supplemental Figure S1.tif]
